# Editorial: Genetics, breeding and engineering to enhance oil quality and yield

**DOI:** 10.3389/fpls.2023.1265897

**Published:** 2023-08-04

**Authors:** Hongbo Chao, Aruna Kilaru, Liezhao Liu

**Affiliations:** ^1^School of Agricultural Sciences, Zhengzhou University, Zhengzhou, China; ^2^Department of Biological Sciences, East Tennessee State University, Johnson City, TN, United States; ^3^College of Agronomy and Biotechnology, Southwest University, Beibei, Chongqing, China

**Keywords:** oil content, oil quality, oil yield, oil crop, genetic improvement

Considering our rapidly increasing global population, the demand for food and edible oil continues to surge. Specifically, the rise in demand for vegetable oils, primarily composed of the storage lipid triacylglycerol, has driven extensive research efforts to enhance oil content and improve oil quality in oilseed crops. The quality of oil crops is important to consumers as they directly contribute to human health by providing essential nutrients along with oil. Vegetable oil quality is determined by its lipid category and fatty acid composition, while additional nutrients like vitamins, minerals, and bioactive compounds influence seed quality, nutritional value, and processing characteristics of oil crops.

Given the growing consumption of high-quality vegetable oils, substantial research has been directed towards enhancing oil content and quality over the years. Various approaches, including conventional breeding, molecular-assisted breeding, and targeted genetic manipulation, have been employed to achieve these goals. This Research Topic compiles a collection of ten articles that focus on increasing oil content and improving oil quality, ultimately deepening our understanding of the genetic control of these traits.

Key areas explored in these articles include:

## Enhancing oil content

Increasing oil content is crucial for ensuring a steady and safe supply of global edible vegetable oil. The identification of important and stable quantitative trait loci (QTLs) sheds light on the genetic basis of oil content and provides a foundation for marker-assisted selection to increase oil content (Zhao et al.). Moreover, candidate genes identified through this research offer valuable genetic resources and targets for improving oil content via targeted genetic manipulation, such as transgenic and gene editing technologies (Xiao et al.; Zhang et al.).

## Improving vegetable oil quality

Attention is also being given to enhancing the quality of vegetable oil. Fatty acid composition plays a pivotal role in determining oil quality. The development of molecular markers associated with fatty acid components facilitates genetic improvements in oil crop quality (Li et al.). Additionally, identifying important functional genes provides genetic resources for cultivating plant oils with specific fatty acid components through metabolic engineering (Behera et al.). Balancing high plant productivity with improved oil quality remains a challenge, as there is often a negative correlation between yield and quality traits in oil crops, such as between flavonoid content and oil content. Understanding the inheritance and regulation of quality traits could lead to further advancements in both yield and quality (Guan et al.).

## Exploration through multiple means or omics

The search for essential genes related to oil content and quality improvement is conducted through various methods or omics. The verification of important homologous genes provides insights into their potential application for improving oil content and quality in oil crops (Behera et al.; Shen et al.; Lin et al.). Additionally, genome-wide data and gene function annotation enable the efficient mining of crucial functional genes (Cheng et al.; Yao et al.).

The research in this Research Topic encompasses various oil crops, such as rapeseed, peanut, sesame, oil palm, and avocado. This diversity of species provides a comprehensive understanding of the research progress in oil crops. Notably, the methodologies employed in these studies are equally diverse and offer a rich repository of references for oil crop research. Altogether, this work deepens our comprehension of the genetic control of oil content and quality in oil crops, paving the way for further genetic improvements in the future.

## Author contributions

HC: Conceptualization, Data curation, Funding acquisition, Writing – original draft, Writing – review & editing. AK: Conceptualization, Data curation, Formal Analysis, Funding acquisition, Supervision, Writing – review & editing. LL: Conceptualization, Supervision, Writing – review & editing.

